# Impact of Physical Exercise on Growth and Progression of Cancer in Rodents—A Systematic Review and Meta-Analysis

**DOI:** 10.3389/fonc.2019.00035

**Published:** 2019-02-05

**Authors:** Robert-Christopher Karl-Richard Eschke, Amit Lampit, Alexander Schenk, Florian Javelle, Karen Steindorf, Patrick Diel, Wilhelm Bloch, Philipp Zimmer

**Affiliations:** ^1^Department for Molecular and Cellular Sports Medicine, German Sport University Cologne, Cologne, Germany; ^2^Department of Psychiatry, The University of Melbourne, Melbourne, VIC, Australia; ^3^Department of Neurology, Charité–Universitätsmedizin Berlin, Berlin, Germany; ^4^Division of Physical Activity, Prevention and Cancer, German Cancer Research Center, Heidelberg, Germany

**Keywords:** physical exercise, tumor growth, cancer, training initiation, rodent models

## Abstract

**Background:** Physical exercise is suspected to reduce cancer risk and mortality. So far, little is known about the underlying mechanisms. Although limited, murine models represent a promising attempt in order to gain knowledge in this field.

**Objective:** A systematic review and meta-analysis examining various treatment protocols was conducted in order to determine the impact of exercise on tumor growth in rodents.

**Methods:** PubMed, Google scholar and System for information on Gray literature in Europe were screened from inception to October 2017. Risk of bias within individual studies was assessed using the Office of Health Assessment and Translation risk of bias rating tool for human and animal trials. The effect of exercise on tumor growth over and above non-exercise control was pooled using random-effects model. Subgroup analyses were conducted to identify potential moderators.

**Results:** The quality of the included 17 articles ranged between “probably low” and “high risk of bias.” A significant reduction in tumor growth in exercising animals compared to controls was detected (Hedges' *g* = −0.40; 95% CI −0.66 to −0.14, *p* < 0.01) with between-study heterogeneity (τ^2^ = 0.217, *I*^2^ = 70.28%, *p* < 0.001). The heterogeneity was partially explained by three moderators representing the in-between group differences of “maximum daily exercise” *R*^2^ = 33% (*p* < 0.01), “type of cancer administration” *R*^2^ = 28% (*p* < 0.05), and “training initiation” *R*^2^ = 27% (*p* < 0.05).

**Conclusion:** This meta-analysis suggests that physical exercise leads to reduction of tumor size in rodents. Since “maximum daily exercise” was found to have at least modest impact on tumor growth, more clinical trials investigating dose-response relationships are needed.

## Introduction

### Rationale

Various fields of health science progressively incorporated the idea that lifestyle factors, such as physical activity could have a greater impact on the general health status than previously believed. Primarily, the interest in physical activity, defined as any bodily movement produced by skeletal muscles that results in energy expenditure (1), was solemnly known to cause positive effects in weight management and reduced risks of suffering from cardiovascular disease, hypertension and type 2 diabetes (2). In the past two decades however, cancer research has also become a considerable target area for the influence of lifestyle changes and physical activity. The number of yearly publications concerning the impact of those variables have nearly doubled in the past 10 years (3).

Observational studies have shown that an increased level of activity are associated with a lower risk for different types of cancer (4, 5). Both, pre- and post- diagnosis physical activity is further associated with reduced cancer-specific and overall mortality in patients suffering from colorectal-, breast-, and prostate cancer (6–8). Besides maintenance or improvements in physical capacity, exercise interventions have proven to reduce frequently observed side effects of cancer diseases and their medical treatments such as fatigue, polyneuropathies, depressions, lymphedema, etc. (7, 9–11). Consequently, a vast body of literature suggests that exercise interventions during and after medical treatment improve quality of life in patients with cancer (12).

Despite the thriving amount of human trials showing positive effects of physical exercise on cancer related outcomes, the knowledge about underlying modes of actions is still limited. However, the understanding of the biological underpinnings is urgently needed to improve general and specific exercise recommendations. Several biological factors concerning the positive influence of regular exercise on cancer development, progress and a reduction of side effects have been investigated for causality. These factors mainly include an exercise-induced reduction of chronic systemic inflammation, activation of the host tumor defense (e.g., by mobilizing and activating tumor competitive immune cells) and alterations of (sexual and metabolic) hormonal and growth factor signaling (13, 14).

The relatively low evidence for key biological mechanisms which is derived from human trials is mainly based on the properties of these studies. Firstly, human studies are very divers due to the heterogeneity of subjects and tumor characteristics. Secondly, there are several methodological challenges, such as difficulties to standardize and control exercise interventions and restricted access to biological material (e.g., tumor tissue). Simultaneously, studies looking at various modes of actions have been published trying to identify the underlying mechanisms in rodents. Clearly, animal models represent great limitations as well. However, the ability to standardize and control the research set up as well as the aptitude to study the pathways underlying human cancer in a complex organisms, justify some of the draw backs of these weaknesses (15).

### Objectives

Against this backdrop, the aim of this research is to identify and make use of the lowest common denominator from the available literature in order to compose a meta-analysis. Herewith, we want to elucidate the current base of knowledge on the impact of physical activity on cancer growth and progression in rodents.

## Methods

This systematic review and meta-analysis follows the Preferred Reporting Items for Systematic Reviews and Meta-Analyses (PRISMA) guidelines (16, 17). The PRISMA checklist is provided as supplement.

### Literature Source and Study Selection

We searched PubMed, Google scholar and SIGLE (System for information on Gray literature in Europe) from inception to October 2017 using the following search string: ((((((((((((exercise) OR physical activity) OR running) OR swimming) OR wheel running) OR treadmill running) OR high intensity) OR low intensity) OR moderate intensity) AND (((((((((((neoplasm) OR breast cancer) OR mammary tumor) OR liver cancer) OR prostate cancer) OR lymphocytes) OR malignancy) OR walker 256) OR morris hepatoma) OR lewis lung carcinoma) OR nitrosomethylurea) AND (((((((odentia) OR mice) OR rats) OR murines) OR rodents) OR animals) OR animal testing) OR animal models) NOT human) OR human trials) AND (((((tumor burden) OR tumor volume) OR tumor weight) OR tumor growth) OR tumor weight per animal). The search string consisted of MeSH-, general-, and rodent specific search terms. A combination of the terms was used in order to find the most relevant articles using indexed and non-indexed terminologies. A detailed search strategy is shown in Table 1. Additional publications were obtained from reference lists of potential eligible articles.

**Table 1:** Electronic data base search strategy.

**Electronic data base search strategy “*****e.g.: PubMed.gov*****”**		
**MeSH terms**	**General terms**	**Rodent specific research terms**
Exercise	Physical activity, running, swimming	Wheel running, treadmill running, high intensity, low intensity, moderate intensity
Neoplasm	Breast cancer, mammary tumor, liver cancer, prostate cancer, lymphocytes, malignancy	Walker 256, morris hepatoma, lewis lung carcinoma, nitrosomethylurea
Rodentia	Mice, rats, murines	Rodents, animals, animal testing, animal models
Tumor burden	Tumor volume, tumor weight, tumor growth	Tumor weight per animal
Human	Human trials	–
Search string:		
((((((((((((exercise) OR physical activity) OR running) OR swimming) OR wheel running) OR treadmill running) OR high intensity) OR low intensity) OR moderate intensity) AND (((((((((((neoplasm) OR breast cancer) OR mammary tumor) OR liver cancer) OR prostate cancer) OR lymphocytes) OR malignancy) OR walker 256) OR morris hepatoma) OR lewis lung carcinoma) OR nitrosomethylurea) AND (((((((rodentia) OR mice) OR rats) OR murines) OR rodents) OR animals) OR animal testing) OR animal models) NOT Human) OR human trials) AND (((((tumor burden) OR tumor volume) OR tumor weight) OR tumor growth) OR tumor weight per animal)		

### Inclusion Criteria

Eligible studies were randomized animal trials investigating the influence of physical activity on cancer growth or size, published in English, German or Dutch. No limits were set for year of publication, duration of trials, type of cancer, spontaneous or planned tumor development, or type of exercise treatment protocols.

Specifically, we included studies that (1) investigated tumor growth in mice or rats (2) used one of the following three outcome measures as dependent value: (a) tumor volume in cubic millimeter/cubic centimeter (mm3/ cm3), (b) tumor weight in gram (g) or (c) tumor weight in milligram per animal in gram (mg ^*^ animal(g)^−1^); (3) had a sedentary control or sham intervention group treated precisely as the experimental group, with the only difference in the exercise component; (4) either had both groups inoculated with tumor cells or none; (5) had voluntary or forced exercise programs including swimming, or treadmill running.

### Exclusion Criteria

Studies were excluded if they were (1) trials performing tests on humans; (2) trials which only had one of the compared groups inoculated with cancer cells; (3) trials that combined exercise training with any kind of other treatment option, e.g., dietary supplementation; (4) reported data were insufficient to include in a meta-analysis.

### Implementation of Search

Two reviewers independently performed the computerized literature search. The first step was screening based on title, followed by screening by abstract. The full-text versions of potentially eligible studies were assessed against the eligibly criteria. Disagreements between the researchers concerning study selection or quality assessment were discussed and consensus was reached.

### Quality Assessment of Included Studies

Internal validity of the included studies was assessed by C.E and A.S with the “Office of health assessment and translation risk of bias rating tool”—for human and animal trials (OHAT) (18). The OHAT for animal trials contains 11 Risk-of-bias questions that cover six different fields of biases including selection, confounding, performance, attrition/exclusion, detection, and selective reporting bias. Eight of the eleven questions can be answered using one of four predefined answer choices that enable the researcher to categorize and quantify the outcome. The choice of answers are (1) “definitely low risk of bias”; (2) “probably low risk of bias”; (3) “probably high risk of bias”; and (4) “definitely high risk of bias.” The remaining three questions are polar questions. (1) Were statistical methods appropriate? (2) Did researchers adhere to the study protocol? (3) Did the design or analysis account for important confounding variables in experimental studies (18)? If any of the questions are answered with “no,” the reason must be elaborated on and an explanation must be given. Studies were excluded from this review if they had an average rating of “definitely high risk of bias” and/or if there was substantial evidence that the studies showed threats to internal validity.

### Outcome Measures and Data Extraction

Following the quality assessment we extracted the dependent values from (1) tumor weight in gram; (2) tumor weight per animal; or (3) tumor volume. For the analysis the researchers were only able to extract the post-treatment data of each group. From these values the mean (M) and standard error of the mean (SEM) or standard deviation (SD) were obtained. When the researchers encountered that a study failed to report data they contacted the lead author of the article, in order to retrieve the missing data. Additional data concerning study size, type of study, study subjects, type of cancer, experimental protocol, duration of the study, and general outcome mentioned in the study was recorded by the researchers. After the extraction the included data was peer-reviewed and confirmed by the senior researcher.

### Data Analysis

The overall agreement between the researchers was calculated using the kappa statistic (19).

The majority of studies used in this review provided outcomes as M and SEM. We calculated standardized means difference (SMD) between exercise and control groups within each study as Hedges' g. Negative SMDs represent a reduction in tumor size and thus a benefit for exercises over control. Hedges' g values of 0.2, 0.5, and 0.8 were interpreted as small, moderate and large effect sizes, respectively. Pooling of outcomes across studies was done using a random-effects model. Between-study heterogeneity was quantified using τ^2^ (which is the variance of true effects) and assessed using the *I*^2^ statistic, which provides the proportion of between-study variance over total observed variance. *I*^2^ value of 75% was considered to be large, 50% moderate and 25% a low proportion of between-study (non-random) heterogeneity (20). Investigations of heterogeneity were performed using mixed-effects subgroup analyses based on key study-level moderators. Small-study effect (“publication bias”) was assessed by visually inspecting funnel plots of SMD vs. standard error (21). When at least 10 studies were available for analysis, asymmetry was formally tested using Egger's test of the intercept (22). If evidence for asymmetry was found (*p* < 0.1 on the Egger's test), the Duval and Tweedie trim and fill method was used to quantify the magnitude of small study effect and to adjust it (23). All analyses were conducted using Comprehensive Meta-Analysis Version 3.

All but two articles incorporated only one study each for the Meta-analysis. Yan and Demars (24) and Westerlind et al. (25) on the other hand, described a methodological process where two independent studies were set up. The subdivided articles were compiled of two sets of independent subject populations. The differences found in between the methodology of the studies were the amount and the way of administration of cancer cell inoculation. Four articles reported more than one of the three outcome measures and were grouped accordingly. With this process the researchers ensured that the data reported was not included as an entirely different publication. Herewith an overestimation of the total weight of the included studies and the overall adjusted effect size was preempted.

## Results

### Study Selection

A total of 572 publications were identified through an electronic database search and cross referencing. After duplicate removal 428 publications were screened by title. Here, 287 papers were excluded because they did not reflect the research question. The remaining 141 publications were screened by the abstract, which lead to the further exclusion of 47 studies because they did not meet inclusion and exclusion criteria. From the residual amount of 94 publications, the full text versions were retrieved and read. Sixty-nine publications were excluded since none of the outcome measures needed for this meta-analysis were reported. Eight articles were excluded because they mentioned a reduction in tumor size or weight in their methods, however, only reported the outcomes in graphs or did not report numerical outcomes (26–33). We contacted the corresponding authors of these studies, but since none fully provided the requested data, all of these studies were excluded from the meta-analysis. This left 17 articles for the final analysis.

**Figure 1:**
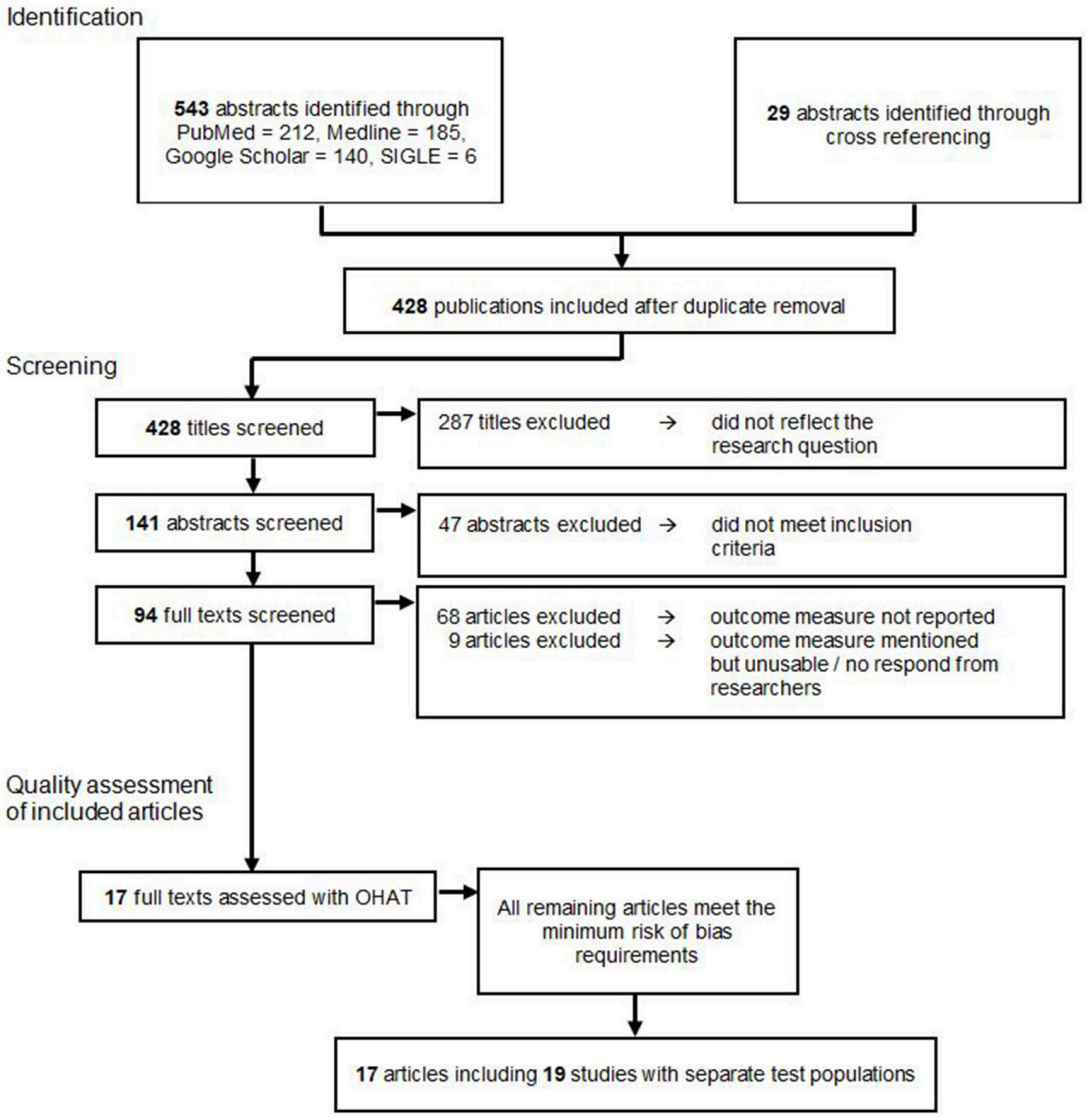
Study selection flow chart according to the PRISMA guidelines.

Ultimately, these 17 publications were examined with the OHAT risk of bias tool. All of the remaining articles met the minimum risk of bias requirements and could therefore be included in the descriptive and this meta-analysis as shown in Figure 1.

Some publications reported results for more than one of the dependent values investigated in this review. Other publications reported two separate studies in one article, this lead to the inclusion of studies in more than one of the meta-analytical computations.

### Interrater Agreement and Quality Assessment of Included Articles

Table 2 shows the studies graded by the authors. All studies scored “probably high risk of bias” in the areas of bias due to group allocation concealment, blinding of the research personnel and blinded outcome assessors. The average outcome of the analysis ranged between “probably high risk of bias” and “probably low risk of bias” (see Table 2). Because none of the evaluated publications scored “definitely high risk of bias” in the overall rating, all selected studies were included in this review.

**Table 2:** OHAT Risk of Bias heat map for randomized controlled trials investigating tumor size in exercised rodents.

**Domains based on (****18****)**		**Tumor weight in gram**	**de Lima et al. (34)**	**de Lima et al. (35)**	**Faustino-Rocha et al. (36)**	**Salomão et al., (37)**	**Shewchuk et al. (38)**	**Tsai et al. (39)**	**Whittal and Parkhouse (40)**	**Whittal-Strange et al. (41)**	**Zhang et al. (42)**	**Tumor weight in volume**	**Almeida et al. (43)**	**Aveseh et al. (44)**	**Bacurau et al. (45)**	**Zhu et al. (46)**	**Tumor weight per animal**	**Gueritat et al. (47)**	**Steiner et al. (48)**	**Yan and Demars (24)**	**Westerlind et al. (25)**
Selection bias	Q1a: Was the administered dose of exposure level adequately randomized?		**–**	**–**	**+**	**–**	**+**	**+**	**+**	**+**	**–**		–	+	+	+		–	+	**+**	–
	Q1b: Was allocation to study groups adequately concealed?		**–**	**–**	**–**	**–**	**–**	**–**	**–**	**–**	**–**		–	–	–	–		–	–	**–**	–
Performance Bias	Q2a: Were experimental conditions identical across study groups?		**+**	**+**	**++**	**++**	**++**	**++**	**++**	**++**	**++**		+	+	+	++		+	++	**++**	++
	Q2b: Were the research personnel and human subjects blinded to the study group?		**–**	**–**	**–**	**–**	**–**	**–**	**–**	**–**	**–**		– –	–	–	–		–	–	**–**	–
Attrition/Exclusion Bias	Q3: Were outcome data complete without attrition or exclusion from analysis?		**–**	**–**	**++**	**–**	**–**	**++**	**++**	**++**	**–**		+	++	++	++		–	++	**++**	–
Detection bias	Q4a: Can we be confident in the exposure characterization?		**++**	**++**	**++**	**++**	**++**	**++**	**++**	**++**	**++**		++	++	++	++		++	++	**++**	++
	Q4b: Can we be confident in the outcome assessment?		**–**	**–**	**–**	**–**	**–**	**–**	**–**	**–**	**–**		–	–	–	–		–	–	**+**	–
Selective Reporting Bias?	Q5: Were all measured outcomes reported?		**++**	**++**	**++**	**++**	**++**	**++**	**++**	**++**	**++**		+	++	++	++		++	++	**++**	++
**Average score**			**–**	**–**	**+**	**–**	**–**	**+**	**+**	**+**	**+**		–	+	+	+		–	+	+	+
Other Bias	Were statistical methods appropriate?		**Y**	**Y**	**Y**	**Y**	**Y**	**Y**	**Y**	**Y**	**Y**		**Y**	**Y**	**Y**	**Y**		**Y**	**Y**	**Y**	**Y**
	Did researchers adhere to the study protocol?		**Y**	**Y**	**Y**	**Y**	**Y**	**Y**	**Y**	**Y**	**Y**		**Y**	**Y**	**Y**	**Y**		**Y**	**Y**	**Y**	**Y**
	Did the study design or analysis account for important confounding variables in experimental studies?		**Y**	**Y**	**Y**	**Y**	**Y**	**Y**	**Y**	**Y**	**Y**		**Y**	**Y**	**Y**	**Y**		**Y**	**Y**	**Y**	**Y**

*Based on the work of Wikoff et al. (49); ++ definitely low risk of bias (dark green); + probably low risk of bias (light green); – probably high risk of bias (light red); – – definitely high risk of bias (dark red)*.

### Publication Bias

**Figure 2:**
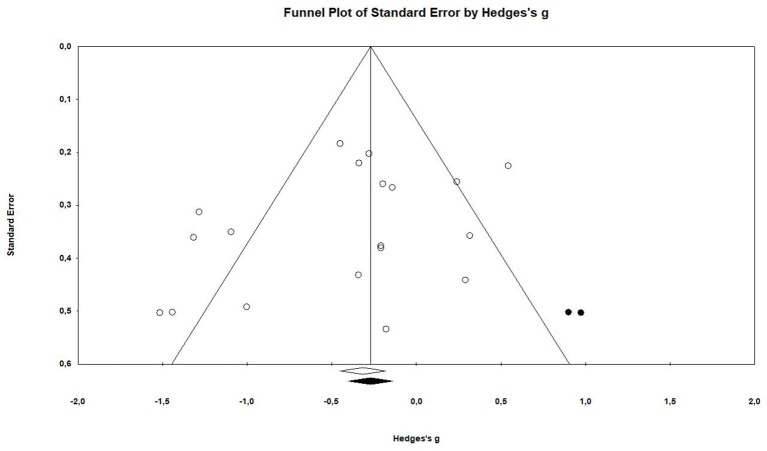
Funnel Plot of standard error against hedges' g after Duval and Tweedie's trim and fill.

The examination of the funnel plot revealed an accumulation of small studies in the lower left corner. Further analysis showed that there was a minor small study bias that showed to be statistically significant according to the Egger's regression (Intercept = −1.97; 95% CI: −4.67 to 0.73; *p* = 0.07; see Table 3).The adjustment from the Duval and Tweedie's trim and fill method suggested that two studies would need to be filled in on the right side of the scatter plot (see Figure 2). This correction changes the effect size by roughly 23%, however it does not influence the confidence interval in a way that it crosses zero (Hedges' *g* = −0.31, 95% CI −0.58 to −0.04).

**Table 3:** Publication bias assessment of the Egger's regression intercepts calculation.

**Egger's regression intercept**	
Intercept	−1.96675
Standard error	1.27892
95% CI lower limit(2-tailed)	−4.66504
95% CI upper limit(2-tailed)	0.73154
*t*-value	1.53782
Degrees of freedom	17
*P*-value (1 tailed)	0.07125
*P*-value (2 tailed)	0.14250

### Sensitivity Analysis

**Figure 3:**
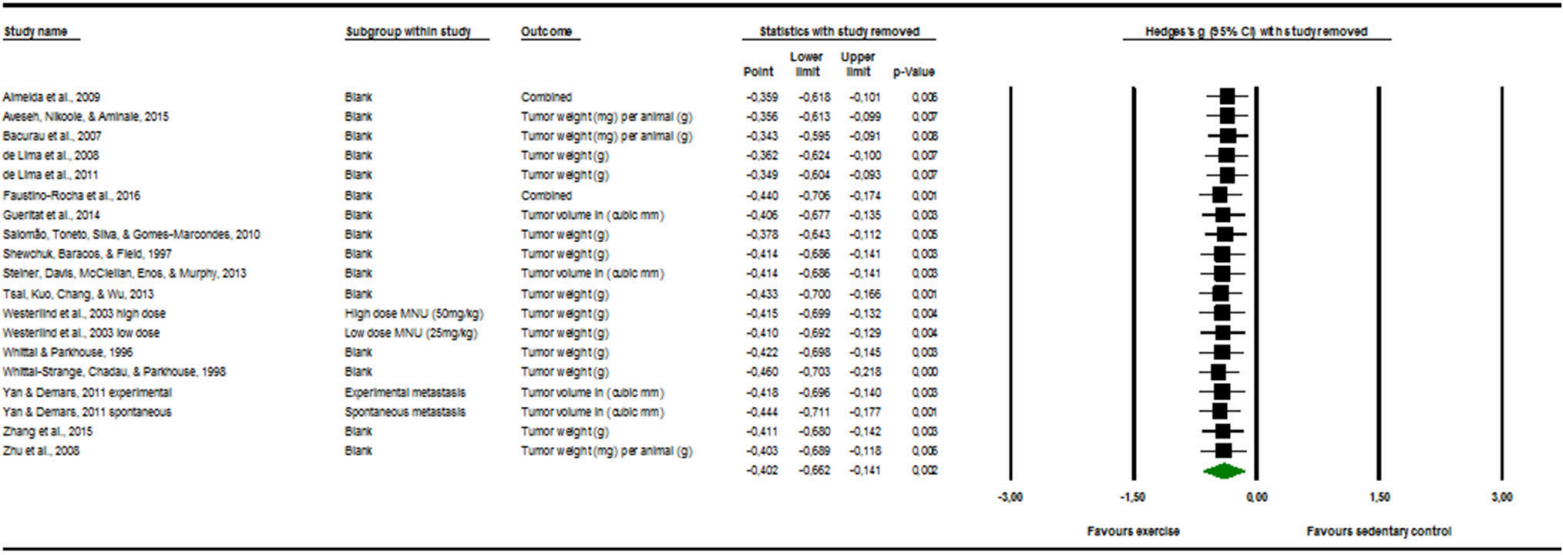
Sensitivity analysis “one study removed” function, of the “comprehensive meta-analysis” program.

The visual inspection of Figure 3 below displayed no evident outlier. This means that it is probable that one study alone could not substantially skew or drive the Hedges' g in either one of the direction.

### Study Characteristics

A total of 17 articles, including 19 separate studies, investigated the weight, and size of tumors in rodents after a controlled exercise regime. The summary of the study characteristics presented in the Table 4, shows the data of 918 assessed animals.

**Table 4:** Summary of studies included in the meta-analysis.

**Summary table**		**Method**							**Results**					
**No**.	**Studies included**	***n***		**Rodent model**	**Tumor type/induction model**	**Exercise protocol**			**Measure**	**Tumor growth progression data**				
		**Ex**	**C**			**Exercise modality**	**Execise prescription (max.)**	**Exercise initiation**		***M***	***SEM***	***M***	***SEM***	**Trend**
1	Almeida et al. (43)	8	11	Male Swiss mice (49 days)	Subcutaneous inoculation of 2 × 10^6^ Ehrlich tumor cells in 0.05 ml in the dorsal area of animals	Swimming with progressive loads	60 min/day; 5 days/weeks	4 weeks before and 2 weeks after inoculation	Tumor weight [mg] per animal [g]	0.180	0.050	0.550	0.100	↓
	Almeida et al. (43)	8	11	Male Swiss mice (49 days)	Subcutaneous inoculation of 2 × 10^6^ Ehrlich tumor cells in 0.05 ml in the dorsal area of animals	Swimming with progressive loads	60 min/d; 5 days/weeks	4 weeks before and 2 weeks after inoculation	Tumor size in [mm3]	0.110	0.030	0.480	0.100	↓
2	Aveseh et al. (44)	9	10	Female BALB/c mice (35 days)	Subcutaneous injection of 1.2 × 10^6^ MC4-L2 cells into right dorsal mammary fat pad	Treadmill running	55 min/d; 7 days/weeks	4 weeks after inoculation for 7 weeks	Tumor weight [mg] per animal [g]	28.500	0.619	31.800	0.298	↓
3	Bacurau et al. (45)	24	24	Male Wistar rats (56 days)	Subcutaneous inoculation of 1 ml sterile suspension of 2 × 10^7^ Walker 256 tumor cells in right flank	Treadmill running	30 min/d; 5 days/weeks	8 weeks before and 2 weeks after inoculation	Tumor weight [mg] per animal [g]	22.200	1.800	38.800	3.200	↓
4	de Lima et al. (34)	18	18	Male Wistar rats (60 days)	Subcutaneous inoculation of 1 ml Walker 256 tumor cells 2 × 107 cells/ml in right flank	Jumping in water	5 min/d; 4 day/weeks	6 weeks before inoculation 2 weeks after	Tumor weight [g]	16.50	1.600	25.100	2.000	↓
5	de Lima et al. (35)	18	18	Male Wistar rats (70 days)	Subcutaneous inoculation of 1 ml Walker 256 tumor cells 2 × 107 cells/ml suspension in right flank	Jumping in water	5 min/d; 4 days/weeks	6 weeks before inoculation 2 weeks after	Tumor weight [g]	17.03	1.190	24.300	1.350	↓
6	Faustino-Rocha et al. (36)	15	15	Female Sprague-Dawley rats (49 days)	Intraperitoneal administration of 50 mg/kg body weight of N-methyl-N-nitrosourea	Treadmill running	60 min/d; 5 days/weeks	Right after inoculation for 35 weeks	Tumor size in [mm3]	7870.00	2820.000	4880.000	1930.000	↗
	Faustino-Rocha et al. (36)	15	15	Female Sprague-Dawley rats (49 days)	Intraperitoneal administration of 50 mg/kg body weight of N-methyl-N-nitrosourea	Treadmill running	60 min/d; 5 days/weeks	Right after inoculation for 35 weeks	Tumor weight [g]	8.310	2.820	5.150	2.040	↗
7	Gueritat et al. (47)	10	10	Male Copenhagen rats (70–84d)	Subcutaneous abdominal tumor fragment implant (20 mg), Dunning AT1	Treadmill running	60 min/d; 5 days/weeks	15 days post-implantation for 4 weeks	Tumor size in [mm3]	3610.00	2280.000	6050.000	2040.000	↙
8	Salomão et al. (37)	8	9	Male Wistar rats (21 days)	Subcutaneous inoculation of 1 ml Walker 256 tumor cells 0,25 × 10^6^ cells/ml in right flank	Light swim training	45 min/d; 5 days/weeks	60 days before and 21 days after inoculation	Tumor weight [g]	40.00	5.500	57.500	5.800	↓
9	Shewchuk et al. (38)	16	11	Female Sprague-Dawley rats (59 days)	Subcutaneous injection of 20 μl Morris Hepatoma 7777 tumor tissue	Swim training	180 min/d; 6 days/weeks	Right after inoculation for 14 days	Tumor weight [g]	8.800	0.500	9.400	1.100	↙
10	Steiner et al. (48)	12	15	Female C3(1)/SV40Tag mice (28 days)	Spontaneous tumor detection	Voluntary wheel running	N.A.	Continuous access to running wheels	Tumor size in [mm3]	3581.60	705.600	4190.800	797.900	↙
11	Tsai et al. (39)	10	9	Male C57BL/6 Mice (56 days)	Subcutaneous inoculation with 5 × 105 Lewis lung carcinoma cells	Treadmill running	60 min/d; 5 days/weeks	1 weeks after inoculation for 4 weeks	Tumor weight [g]	4.640	1.120	3.700	0.830	↗
12	Westerlind et al. (25) high dose	46	51	Female Sprague-Dawley rats (20 days)	Intraperitoneal administration of 50 mg/kg body weight of 1-methyl 1-nitrosourea (MNU)	Treadmill running	30 min/d; 5 days/weeks	Right after inoculation 2,4,6, or 8 f weeks	Tumor weight [mg] per animal [g]	7.270	2.400	10.020	2.650	↙
13	Westerlind et al. (25) low dose	32	57	Female Sprague-Dawley rats (20 days)	Intraperitoneal administration of 25 mg/kg body weight of 1-methyl 1-nitrosourea (MNU)	Treadmill running	30 min/d; 5 days/weeks	Right after inoculation for 2,4,6, or 8 weeks	Tumor weight [mg] per animal [g]	1.173	0.267	2.580	0.519	↙
14	Whittal and Parkhouse (40)	26	29	Female Sprague-Dawley rats (21 days)	Intraperitoneal administration of 50 mg/kg body weight of nitrosomethylurea (NMU)	Treadmill running	60 min/d; 5 days/weeks	39 days before inoculation	Tumor weight [g]	2.100	0.650	2.600	0.670	↙
15	Whittal-Strange et al. (41)	40	40	Female Sprague-Dawley rats (21 days)	Intraperitoneal administration of 37.5 mg/kg body weight of nitrosomethylurea (NMU)	Treadmill running	60 min/d; 5 days/weeks	39 days before inoculation	Tumor weight [g]	3.200	0.740	1.200	0.340	↑
16	Yan and Demars (24) experimental metastasis	30	30	Male C57BL/6 mice (21 days)	Subcutaneous injection of Lewis Lung Carcinoma (2.5 x 10^5^/50 μl/mouse) in the lower dorsal region	Voluntary wheel running	N.A.	9 weeks before inoculation and cont. for 2 weeks after removal	Tumor size in [mm3]	0.010	0.001	0.012	0.001	↙
17	Yan and Demars (24) spontaneous metastasis	30	28	Male C57BL/6 mice (21 days)	Intravenous injection of B16BL/6 cells (0.75 × 10^5^/200 μl/mouse) through lateral tail vein	Voluntary wheel running	N.A.	9 weeks before inoculation and cont. for 2 weeks	Tumor size in [mm3]	2.136	0.171	1.928	0.138	↗
18	Zhang et al. (42)	6	6	Male C57BL/6 Mice (42 days)	Subcutaneous injection of 5 × 10^6^ Hepa1-6 cells +3 × 10^6^ Hepa1-6-green fluorescent protein cells	Voluntary swimming	8 min/day;5 days/weeks	3 weeks before and 6 weeks after inoculation	Tumor weight [g]	2.570	0.680	3.190	1.120	↙
19	Zhu et al. (46)	60	60	Female Sprague-Dawley rats (20 days)	Intraperitoneal administration of 50 mg/kg body weight of 1-methyl 1-nitrosourea (MNU)	Voluntary wheel running	N.A.	7 days after inoculation for 8 weeks	Tumor weight [mg] per animal [g]	0.620	0.140	1.160	0.210	↓
		441	477											
Total number subjects		918												

*Summary of studies that displayed tumor size in tumor weight. n, number of subjects; Ex, exercise group; C, sedentary control group; d, day(s);weeks, week(s); M, Mean; S.E.M, standard error of the mean; No. , Number; ?, slight increase in tumor size; ?, slight decrease in tumor size; ↑, increase in tumor size; ↓, decrease in tumor size*.

#### Tumor Weight in Grams

Under various exercise treatment conditions, the final tumor weight in grams was assessed in a total of nine studies (34–42). In these controlled trials two species, two subtypes of species and different age ranges were observed. Female *Sprague-Dawley rats* were most commonly utilized (38, 40, 41), followed by male *Wistar rats* (34, 35, 37). Two studies investigated male *C57BL/6 Mice* (39, 42).

The age of the animals varied between 21 and 70 days of age at the beginning of the studies. Tumor types were derived from different mammary (3x Walker 256, 1x N-methyl N-nitrosourea, 2x nitrosomethylurea), two liver (1x Morris hepatoma 7777, 1x Hepa 1-6), and lung carcinoma (Lewis lung carcinoma) cell types.

The exercise modalities were composed of forced treadmill running (36, 39–41), as well as light and moderate swim training, respectively (37, 38). Two studies also included jumping in water as an exercise modality (34, 35). The exercise prescription ranged from 5 to 180 min per day and between 4 to 6 days per week. Similarly, diverse were the exercise initiations in relation to the inoculation time points as well as the durations of the studies. The initiation of exercise programs presented a range from 6 weeks before inoculation until 1 week after inoculation. The duration of the exercise program ranged from 14 days until 35 weeks.

#### Tumor Weight Per Animal

A total of six studies investigated the tumor weight per animal from a total of 392 samples. In these trials two different species were utilized. Three of the six subject groups consisted of female *Sprague-Dawley rats*. The remaining three trials used male *Wistar rats*, male *Swiss*, and female *BALB/c mice*. All of the six tumor models investigated different mammary carcinoma (3x 1-methyl 1-nitrosourea; 1x MC4-L2 human breast cancer; 1x Ehrlich tumor; 1x Walker 256) cell lines.

The exercise modalities consisted mainly of treadmill running (25, 44, 45), however one study focused on voluntary wheel running (46) and another on swimming with progressive loads (43). In the studies that assessed forced exercise, the prescription ranged from 30 to 60 min daily and five to seven times per week. Exercise initiation varied from 8 weeks before until 4 weeks after inoculation. The total duration of training programs diverged from 6 to 10 weeks.

#### Tumor Volume

A total of four studies investigated the size of tumors solely by measuring the volume (24, 47, 48). Two studies additionally looked at the tumor volume among other measures but did not use separate test subjects (36, 43). In total 214 rodent samples could be integrated in the analysis that performed voluntary and forced exercise. These samples were also composed of different species and subspecies. Two trials used rats (1x female *Sprague-Dawley* rats, 1x male *Copenhagen rats*) and the residual four studies incorporated mice as their study subjects (2x male *C57BL/6 mice*, 1x male *Swiss mice*, 1x female *C3(1)/SV40Tag mice*).

In this assortment, tumor models ranged noticeably between the studies. Two of them investigated spontaneous tumor development, whereas one study injected B16BL/6 melanoma cells, and another did not inoculated tumor cells. This study used mice that are by nature prone to tumor development. Another two studies assessed tumor volume of different mammary carcinomas (1x Ehrlich tumor cells, 1x N-methyl-N-nitrosourea). The residual two studies looked at rat prostate cancer (Dunning AT-1) and lung carcinoma (Lewis Lung carcinoma).

The majority of studies examined the effect of forced treadmill running (four studies). The remaining two studies investigated swimming with progressive loads and voluntary wheel running. Exercise prescription ranged from 30 to 60 min daily, five to seven times per week.

In this group, exercise initiation started as early as 8 weeks before tumor inoculation and as late as 4 weeks post-inoculation. The median duration of training was 9 weeks.

### Quantitative Outcome Measures

**Figure 4:**
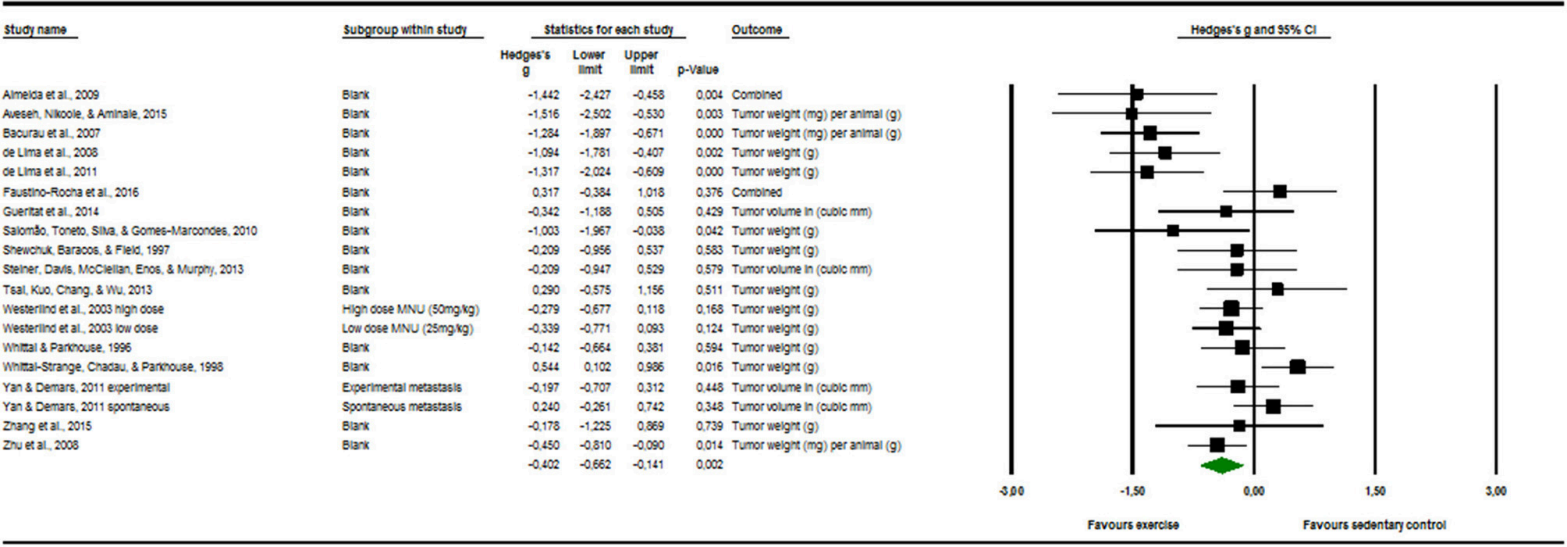
Meta-analysis of tumor software output presenting Hedges's g, 95% CI (confidence interval) and *p*-value of the random effect model of tumor size. The size of the squares and the thickness of the horizontal lines express the weight of the presented study. The total Hedges' g effect size is presented as green diamond. Its width expresses the CI. The vertical line at zero represents no difference in adjusted effect size of exercise on tumor growth. Data on the left from the line stands for a decrease and data on the right stands for an increase of tumor size. The heterogeneity was calculated and was shown to be at the end range of moderate [*I*^2^ = 70.28%; *Q* = 60.57; df(Q) = 18; *p* < 0.00].

Seventeen articles reported outcome measure for tumor size in a total of nineteen studies (*k* = 19). After standardization to a single common measurement, the data was aggregated in the random effect model of the meta-analysis of tumor size. The overall standardized effect of exercise on tumor over and above control was small to moderate and statistically significant, with large between-study heterogeneity (Hedges' *g* = −0.40, 95% CI: −0.66 to −0.14, *p* < 0.01, τ^2^ = 0.217, *I*^2^ = 70.28%, see Figure 4). The funnel plot revealed possible asymmetry toward larger effects in smaller studies (Egger's Intercept = −1.97, *p* = 0.07). A trim and fill analysis suggested the addition of two studies to the right of the funnel; the resulting effect size was about one-quarter smaller, remained however statistically significant, (Hedges' *g* = −0.27, 95% CI −0.40 to −0.13) depicted by the black diamond in Figure 2.

### Investigations of Heterogeneity

#### Grouped by Outcome Measure

**Figure 5:**
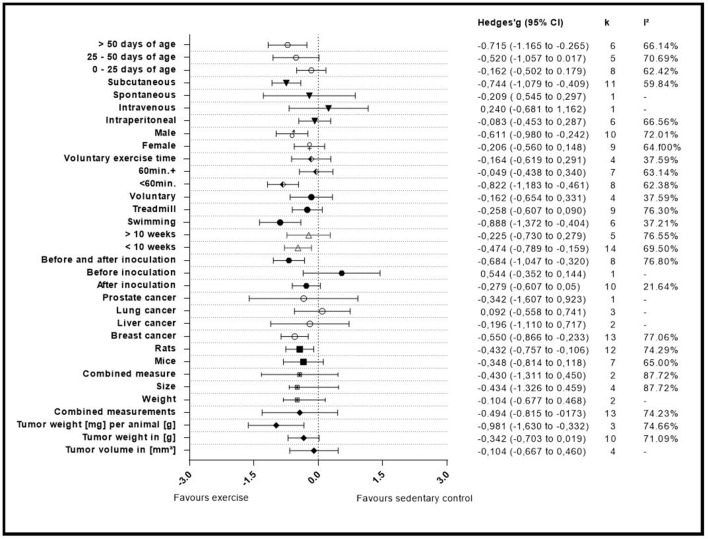
Adjusted effect sizes of the investigated groups tested by the predefined moderators, *k*, number of studies in the subgroup; *I*^2^, measure of heterogeneity.

A summary of the group analyses is shown in Figure 5 and Table 5. Here the first grouped analysis shows the studies that were split into “tumor volume,” “tumor weight in gram,” “tumor weight per animal,” and “Combined.” “Combined” stands for the publications that reported more than one outcome measure at once. The first subgroup represented eight publications and the remaining three subgroups each included three publications. Grouping according to this moderator did not produce statistical significance between the subgroups of studies investigating tumor volume (Hedges' *g* = −0.10, 95% CI −0.67 to −0.46), tumor weight in gram (Hedges' *g* = −0.34, 95% CI −0.70 to 0.02), tumor weight per animal (Hedges' *g* = −0.98, 95% CI −1.63 to −0.33) and combined measurements (Hedges' *g* = −0.43, 95% CI −1.31 to 0.45, Q-between = 4.74, *df* = 2, *p* = 0.09). This relationship was able to accounted for 8% of the differences between the subgroups (*R*^2^ = 0.08).

**Table 5:** Moderator congregated by different covariates.

**Moderator groups**	**Covariates**	**Amount of studies**	**In-between subgroup differences**			**Meta-regression**
			***Q***	**Degrees of freedom (df)**	***p*-Value**	***R*^**2**^ (%)**
Outcome measure	Tumor volume [mm3]	4	4.74	2	0.094	8
	Tumor weight in [g]	10				
	Tumor weight [mg] per animal [g]	3				
	Combined measurement	2				
Measure	Weight	13	1.49	1	0.2219	0
	Seize	4				
	Combined measurement	2				
Type of rodent	Mice	7	0.08	1	0.7730	0
	Rats	12				
Type of cancer	Breast cancer	13	3.26	3	0.354	0
	Liver cancer	2				
	Lung cancer	3				
	Prostate cancer	1				
Training initiation	After inoculation	10	7.12	2	0.0284*	27
	Before inoculation	1				
	Before and after inoculation	8				
Exercise period	< 10 weeks	14	0.67	1	0.4132	0
	>10 weeks	5				
Type of exercise	Swimming	6	5.44	2	0.0659*	17
	Treadmill running	9				
	Voluntary wheel running	4				
Maximum daily exercise	< 60 min	8	9.41	2	0.0091*	33
	60 min+	7				
	Voluntary exercise time	4				
Gender	Female	9	2.42	1	0.120	6
	Male	10				
Type of cancer administration	Intraperitoneal	6	8.85	3	0.031*	28
	Intravenous	1				
	Spontaneous	1				
	Subcutaneous	11				
Age	0–25 days of age	8	3.95	2	0.1388	20
	25–50 days of age	5				
	>50 days of age	6				

*P < 0.05.

#### Grouped by Type of Measure

The second group analysis investigated the in-between group differences between “size,” “weight,” and “combined measure” which were pooled during the meta-analytic computations. The largest subgroup “weight” was represented by thirteen studies. The subgroup “size” consisted of four studies. “Combined measure” was composed of two studies. During this analysis no statistical significant differences of “weight” (Hedges' *g* = −0.10, 95% CI −0.68 to 0.47), “size” (Hedges' *g* = −0.43, 95% CI −1.31 to 0.45), or “combined measure” (Hedges' *g* = −0.43, 95% CI −1.31 to 0.45, Q-between = 1.49, *df* = 1, *p* = 0.22) were found in between the different types of measures used for tumor size. As expected by the high *p*-value there is no relationship that could explain the difference between the subgroups (*R*^2^ = 0.00).

#### Grouped by Type of Rodent

In the following analysis the publications were divided into two subgroups. One group compiled of seven studies, representing mice and the other subgroup representing rats with 12 studies. As in the first grouped analysis this did not produce a significant difference between mice (Hedges' *g* = −0.35, 95% CI −0.81 to 0.12) and rats (Hedges' *g* = −0.43, 95% CI −0.76 to −0.11, Q-between = 0.08, *df* = 1, *p* = 0.77). Accordingly, no relationship was found in-between the subgroup differences of this moderator value *R*^2^ = 0.00.

#### Grouped by Type of Cancer

With this analysis subgroups were assembled according to the type of cancer examined in the individual studies. This meant that the largest subgroup displayed the combined adjusted effect size of thirteen studies (“Breast”). The following two groups (“Liver” and” Lung”) incorporated the effect size from two and three studies, respectively. The last subgroup only consisted of one study that investigated prostate cancer (“Prostate”). When grouped by types of cancer there were no significant differences found between breast cancer (Hedges' *g* = −0.55, 95% CI −0.87 to −0.23), liver cancer (Hedges' *g* = −0.20, 95% CI −1.11 to 0.72), lung cancer (Hedges' *g* = 0.09, 95% CI −0.56 to 0.74) and prostate cancer (Hedges' *g* = −0.34, 95% CI −1.61 to 0.92, Q-between = 3.26, *df* = 3, *p* = 0.35). As well as in the preceded computation no relationship was found between the subgroup differences *R*^2^ = 0.00.

#### Grouped by Initiation of Training

The covariate “Initiation of training” consisted of three subgroups. The studies were grouped by the start of the training regimen which could either be “before inoculation,” “after inoculation,” or “before and after” the inoculation. These subgroups consisted of one, ten and eight studies, respectively. This grouped analysis was the first one to show a significant difference between the subgroups of “after inoculation” (Hedges' *g* = −0.28, 95% CI −0.61 to 0.05), “before inoculation” (Hedges' *g* = 0.54, 95% CI −0.35 to 1.44) and “before and after inoculation” (Hedges' *g* = −0.68, 95% CI −1.05 to 0.32, Q-between = 7.12, *df* = 2, *p* < 0.05). This relationship accounted for 27% of the differences between the subgroups (*R*^2^ = 0.27).

#### Grouped by Exercise Period

Only two subgroups were set up in the covariate “exercise period.” The first subgroup “<10 weeks” consisted of fourteen studies. The second subgroup “>10 weeks” consisted of five studies. Furthermore, the time frame in which the animals were exercised in the relative groups did not present a significant effect between “<10 weeks” (Hedges' *g* = −0.47, 95% CI −0.79 to −0.16) and “>10weeks” (Hedges' *g* = −0.23, 95% CI −0.73 to 0.28, Q-between = 0.67, *df* = 1, *p* = 0.41). Again, as with the grouping for “type of rodent” and “type of cancer,” “exercise period” no relationship was found to explain the differences between the subgroups (*R*^2^ = 0.00).

#### Grouped by Type of Exercise

Type of exercise was the following examined covariate. It was organized into three subgroups “Swimming,” “Treadmill running,” and “Voluntary wheel running.” Six, nine and four studies were included in the subgroups, respectively. The mode of exercise also did not yield a significant in-between subgroup significance as the result showed (Hedges' *g* = −0.89, 95% CI −1.37 to −0.40) for “Swimming,” (Hedges' *g* = −0.26, 95% CI −0.61 to −0.09) for “Treadmill running” and (Hedges' *g* = −0.16, 95% CI −0.65 to 0.33, Q-between = 5.44, *df* = 2, *p* = 0.07) for “Voluntary wheel running.” Through the fact that this difference was nearly significant, the type of exercise was able to explain 17% of the difference between the subgroups (*R*^2^ = 0.17).

#### Grouped by Maximum Daily Exercise

By dividing the studies into subgroups that focused on the maximum prescribed exercise regimen which subjects had to complete, the researchers were able to examine the effect size of the subgroups and the in-between subgroup effect of the covariates. Eight studies provided data for the “<60 min” daily exercise subgroup (<60 min). For the “60 min and more” subgroup (60 min+) seven studies were included. The last subgroup was composed of the data from the studies that investigated voluntary exercise programs and did not report a daily exercise time (Voluntary exercise time). This subgroup provided four studies. The computation showed that there was a statistical significant difference between studies that incorporated “<60 min” of training (Hedges' *g* = −0.82, 95% CI −1.18 to 0.46) compared to those that containing more than 60 min (Hedges' *g* = −0.05, 95% CI −0.44 to 0.34), or voluntary exercise time (Hedges' *g* = −0.16, 95% CI −0.62 to 0.29, Q-between = 9.41, *df* = 2, *p* < 0.01).

This relationship of 33% explains most of the differences between the subgroups (*R*^2^ = 0.33).

#### Grouped by Gender

Gender as moderator divided the data in two subgroups with nine studies in the “Female” and 10 studies in the “Male” group. This computation did not produce an in-between difference between the “Female” (Hedges' *g* = −0.21, 95% CI −0.56 to 0.15) and “Male” (Hedges' *g* = −0.61, 95% CI −0.98 to −0.24, Q-between = 2.42, *df* = 1, *p* = 0.12) subgroups. Although not significant, this relationship explains 6% of the differences between the subgroups (*R*^2^ = 0.06).

#### Grouped by Type of Administration

Various types of administration of the cancer cells were mentioned before. Due to this the researchers decided to setup four subgroups during this analysis. Two subgroups were only composed of one study each (“Intravenous administration” and “Spontaneous cancer development”) while the other two subgroups were represented by 11 and six studies (“Subcutaneous” and “intraperitoneal”), respectively. The type of administration did present a significant subgroup difference between “Intraperitoneal” (Hedges' *g* = −0.08, 95% CI −0.45 to 0.28), Intravenous (Hedges' *g* = −0.24, 95% CI −0.68 to 1.16), Spontaneous (Hedges' *g* = −0.21, 95% CI −0.55 to 0.30), and subcutaneous (Hedges' *g* = −0.74, 95% CI −1.08 to −0.41, Q-between = 8.85, *df* = 3, *p* < 0.05). This relationship accounted for 28% of the difference between the subgroups (*R*^2^ = 0.28).

#### Grouped by Age

In the last grouped analysis the researchers defined three age ranges for the computation of the data. The first subgroup consisted of eight studies that had animals included of “0–25 days of age.” The second subgroup consisted of animals ranging between “25–50 days of age” and included five studies. The last subgroup contained all animals that were older than fifty days (“>50 days of age”), entailing six studies. This analysis was not able to identify significant differences between “0–25 days of age” (Hedges' *g* = −0.16, 95% CI −0.50 to 0.18), “25–50 days of age” (Hedges' *g* = −0.52, 95% CI −1.06 to 0.02) and “>50 days of age” (Hedges' *g* = −0.72, 95% CI −1.17 to −0.27, Q-between = 3.95, *df* = 3, *p* = 0.14). Despite the fact that there was no significance, this analysis explains 20% of the differences between the subgroups (*R*^2^ = 0.20).

## Discussion

The overall results from this meta-analysis suggest that physical activity positively impacts tumor size in rodents. However, these results represent data from very diverse and heterogenic study backgrounds, which limit the expressiveness for individual research designs. This is a general dilemma in the context of exercise-oncology that has already been established by Ashcraft et al. (6). Nevertheless, the majority of the included studies reported an effect as a statistical significant reduction not only in tumor weight (34, 35, 37, 43–45), but also in an increased life span and/or increased latency period (25, 45, 48). This goes in line with an earlier review looking at the effects of physical exercise on experimentally induced mammary carcinogenesis (50).

Interestingly, although reporting an increase in tumor weight and volume in exercised animals, one study reported that the tumors of the exercised animals were less aggressive and that these animals also had an increased latency period (36). Faustino-Rocha and colleagues linked this phenomenon to enhanced blood perfusion initiated by an over-expression of the vascular endothelial growth factor A (VEGF-A). In contrast, Tsai et al. (39) as well as Yan and Demars (24) were not able to report any significant changes of VEGF in their exercised animals compared to the sedentary counterparts of their study. A recent published review shares this contradictory role of VEGF, stating that the mechanisms involved in the regulation of tumor vascularization are very complex. To their opinion, the great variety of tumor vessels need to be further investigated to exploit the underlying mechanisms of growth factor activity. Especially in consideration of the different growth factors such as angiopoietins, platelet derived growth factor (PDGF-B) and transforming growth factor (TGF-ß) (51).

Besides aspects of tumor vascularization, human trials, and murine models have focused on the activation of the innate immune system where research concentrates on the count and measurement of redistributed natural killer (NK-) cells (52). Only one of the included studies in this review investigated the changes of NK-cell count, none examined redistribution. Although Shewchuk et al. (38) were not able to find a statistical significant effect of exercise, it showed a reduction in tumor size and identified a statistical significant increased number of NK-cells in the spleens from exercised animals compared to the sedentary control. This is in line with recent research of Pedersen et al. (29), who showed for the first time that voluntary running suppressed tumor growth and increased intra-tumoral NK-cell numbers. However, the authors also reported alterations of other immune cell numbers in the tumor microenvironment. Therefore, further research is needed to clarify the role and importance of NK-cells in this context.

The question, if there is a probable dose-response relationship between exercise modality and prognosis is an even greater subject of discussion in the literature. Until now it is not clear what kind of exercise type, intensity, period, duration, or onset might be the best to counteract cancer development and progress. Results from human observational trials suggest that individuals reporting the highest activity levels indicate lowest cancer-specific and overall mortality rates. However, these studies are limited mainly because physical activity is assessed by self-reporting. Evidence from prospective RCTs is still lacking.

In terms of the exercise period, this meta-analysis presents data that suggests an exercise period below 10 weeks is superior to an exercise period exceeding 10 weeks, because it showed a better outcome in tumor reduction. This outcome should be interpreted carefully. It needs to be considered that most rodent species develop tumors in later stages of their life's (53). This could mean that the difference between groups could be eliminated with studies that last longer than 10 weeks. On the other hand, studies shorter than 10 weeks could obscure the full potential of exercise. For the latter the results of the meta-regression might show a lead. Although an insignificant in-between group difference is seen, the adjusted effect size for the entry age of animals above 50 days showed a greater tumor reduction than animals below the age of 25 days. As in humans most of the tumor species are typically age related diseases, the transferability of early onset tumor models in rodents seems to be questionable. Nevertheless, our results allow us to make the assumption that there might be a greater effect in the older animals or in a later point of time during a study. Knowing that the average tumor onset in humans is also in later life stages, as seen in the example of colorectal cancer, transferring these results to humans is difficult (54).

Similarly, diverse is the prescription of exercise duration as mirrored in this meta-analysis. This is why the researchers created a moderator value that subdivided the studies only in two groups based on the maximum daily exercise. Here the meta-regression was able to show that daily exercise prescriptions shorter than 60 min reduced the tumor growth significantly compared to exercise prescriptions that were longer than 60 min, or than voluntary wheel running (Supplements Table 4). This finding might be explained by the increased challenge for the immune system due to high training loads and over training (55). Further, the significant difference could be due to the fact that the animals in the voluntary exercise training group stopped exercising when the potential benefit could have been most profound. So far this subject of exercise duration was not presented by any other review and could be a suggestion for further research.

Another interesting finding was that swimming as a choice of exercise showed the greatest effect size from the three exercise models presented in this meta-regression. Although there were no significant in-between group differences, this model was able to explain 17% of the heterogeneity of the data set. This indicates that further investigations on type of training as an effect moderator are needed. To our knowledge there are no studies that compare the effects of different training models, making this area another target for further research. Nevertheless, swim training is controversially discussed in the literature as it puts the animals under a great distress (56).

Training initiation was another moderator that explained a great proportion of the heterogeneity during the meta-regression. It showed that the training “before and after” as well as “after” inoculation significantly exceeded the effects of the groups that only exercised “before” the inoculation/injection process. However, this comparison has two major limitations. While one is investigating a potential preventive effect, the other is rather related to a rehabilitative setting. Thereby, two completely different settings are investigated. Additionally, taking away a potential beneficial environmental factor (deconditioning) during the course of disease may even worsen the later outcome. Nevertheless, the suggestion that the effect of exercise is more related to a direct effect, as opposed to a protection effect, cannot be made. This is because the group representing “before inoculation” merely consisted of one study. However, what can be said is that the combination of training before and after inoculation produced the highest effect size in this group.

In general, exercise modalities seem to differentially influence tumor growth in rodents. The transferability of these findings to the clinical setting is difficult due to many reasons. Firstly, an active lifestyle should be recommended independent of cancer. Therefore, the onset of starting with exercise in humans should ideally be before the occurrence of any chronic disease. Secondly, the interpretation of “exercise” in rodents is critical. In a real life setting mice and rats are nocturnal animals. Putting these animals in small cages, does not display the natural habits of these animals. “Exercise” in these studies might still be less than the actual amount of time animals would usually be active in real life. However, one could argue that *homo sapiens* had also been much more active during their evolution than they are today (especially when compared to the western world lifestyle). Thirdly, some kind of exercise interventions (e.g., swimming) may induce distress in animals. Thereby, the influence of bio-psychological factors cannot be ruled out. Fourthly, tumor growth may not be the most important clinical endpoint, as e.g., the aggressiveness of a tumor displayed by the capability to spread and metastasize can also be a important outcome measure to look at (6, 7). Fifthly, only few rodent studies combined exercise with a standard medical treatment such as chemotherapy, which is common practice in the clinical setting.

Gender as a moderator did not produce any significant in-between group differences. It did however contribute to a small amount to the overall heterogeneity. Additionally, it seems that male animals benefit slightly more from exercise then their female counterparts. This is evident through the circumstance that male animals presented a significant adjusted effect size and female animals did not. Considering the fact that the majority of cancer models were mammary derived this result is not surprising as it has been shown that many mammary cancers are estrogen driven and would exacerbate more in female than in male animals (57).

Presented as second largest group difference, the type of tumor-administration also played an underscored role in the presence of heterogeneity. There seemed to be a significant difference between tumors administered to the intraperitoneal space and subcutaneous injection. Subcutaneous injections presented with a greater adjusted effect size than the residual groups. This was also criticized in a review by Talmadge et al. (58). They stated several limitations of the rodent model such as no incorporation of biological concepts, drug pharmacology, and that neither ascetic nor transferable subcutaneous tumors are predictive of activity for solid tumors. This finding supports the question, if the currently used techniques of administering cancer cells is the most realistic in identifying the potential benefits from exercise on cancer and if the animal model from rodents is the most precise option we have. Adversely, a meta-analysis of Corpet and Pierre (59) showed that rodent models of rats react approximately analogous to colon chemoprevention with some agents and roughly predict an effect in humans.

Although only represented with one study each, this review also entailed two articles reporting results for alternative cancer treatment options which were part of the studies in separate groups (42, 47). The study from Gueritat et al. presented additional result that showed that the consumption of antioxidant supplementation (pomegranate juice) decreased prostate cancer proliferation. The underlying mechanism is described as a regulation of various signaling pathways in prostate tumors. Interestingly, the combination of pomegranate juice with exercise, also hypothesized as antioxidant, did not potentiate the effect. Instead it seemed to blunt the effect of exercise. On the contrary, Zhang et al. (42) investigated the effect of an herbal compound originated from Traditional Chinese Medicine (TCM), that in combination with exercise reduced tumor size. Further, that effect was even greater than exercise alone. Their research showed that the Songyou Yin (SYY) compound statistically significant reduced tumor growth as well as raised the CD4+ to CD8+ ratio. This ratio has been proposed to be a prognostic marker for patient outcome with hepatocellular carcinoma (60).

### Limitations

The most prominent limitation of this work is the lack of reporting standard as well as the non-compliance of researchers to provide necessary data for meta-analysis. In terms of reporting- standard it was observable that in some studies only data from the groups that yielded significant results were published. In other studies results were presented in a manner that they were useless for meta-analytical computations. Some studies left out insignificant values completely or posted only graphs and figures. Even upon request none of the contacted researchers turned over the requested data. Furthermore, the quality of studies presents a significant fraction of risk of bias on the overall outcome and questions its magnitude. Especially the areas concerning “blinding of research personnel,” “outcome assessment,” and “concealment of study group allocation.” Scoring “probably high risk of bias” in these areas only because there was no information given in the articles, calls for a close adherence to standardized randomized controlled trial protocol. Another aspect of limitations was the small sample size of studies included in this meta-analysis. This matter diminishes the meaningfulness and the power of the finding and should be considered by the reader.

Recently it has also been discussed that the enrichment of animal cages can also have an influence on tumor growth (58, 61–63). Interestingly, none of the studies included in this meta-analysis further reported detailed information on animal housing. We can note the following differences in the animal housing. Only one study mentioned the animals bedding and the use of filter-capped polycarbonate cages (36). Salomão et al. (37), reported that the animals were housed in collective cages. Shewchuk et al. (38), reported that animals were contained in wire-mesh cages while Yan et al. (24), on the other hand, used wire-topped plastic boxes for storage of the animals. Lastly, Zhu et al. (46) incorporated solid-bottomed polycarbonate cages for their animals. No correlations or interpretations were made with this variable.

Finally, included studies are hardly comparable due to their differences in study designs. An example is the used rodent diet. Bioactive substances in the diets can influence molecular and cellular events in tumors. Especially dietary estrogens can profoundly influence molecular and cellular event actions on estrogen receptors androgen receptors and estrogen-sensitive genes (64). This is of major importance in tumor models for estrogen or androgen sensitive tumors like breast (65, 66) or prostate cancer (67). However also in non-steroid hormone dependent tumor models effects of such substances in the diet have been described (68). The major source of these dietary estrogens is soy, which is part of the most commercially rodent diets (69). Moreover, also the concentration of these substances varies from batch to batch (69). In the included 17 papers only in 4 papers (24, 25, 37, 46) purified, soy free diets have been used. In all other papers used diets are either based on soy or the authors did not even address the exact composition of the diet. This is a significant limitation regarding the interpretation of the results. Finally, we included only specific cancer types in our search string. We have chosen this attempt in order to focus on the most frequently used cancer models in order to avoid a too heterogeneous mix of entities. Further research may include all existing cancer models. However, results provide a good overview of the current trend, regarding the impact of physical activity in murine cancer research.

## Conclusion

This meta-analysis was performed to identify the lowest common denominator and the general trend of the impact of physical activity on tumor growth and progression. In respect to the encountered limitations the researchers found a significant effect of physical activity on cancer growth presenting itself in the reduction of tumor size in the murine model. A moderate amount of heterogeneity was identified and investigated accordingly. Meta-regression identified that the moderator models of training initiation, maximum daily exercise, and type of cancer administration accounted for the majority of this heterogeneity. As disclosed by Mak et al. (70) the average rate of successful translation from murine cancer to human trials only lies at 8%. Nevertheless, these trials are of immense value to further exploit the mode of actions of physical activity on the growth and progression of cancer in rodents. Fully understanding the mode of actions in the murine model has the potential to become a milestone in unraveling the inscrutable and unyielding character of cancer.

## Author Contributions

PZ, KS, WB, AL, and PD designed research. R-CE, AS, and FJ conducted research. R-CE, FJ, and AL conducted statistical analysis. R-CE, PZ, PD, and AS wrote the paper.

### Conflict of Interest Statement

The authors declare that the research was conducted in the absence of any commercial or financial relationships that could be construed as a potential conflict of interest.
